# Supplementary material for: Node-equivariant message passing for efficient and accurate machine learning interatomic potentials
Source: Chem Sci. 2025 Dec 23;17(7):3793–803. doi: 10.1039/d5sc07248d (PMC12766320; doi:10.1039/d5sc07248d)
Supplement: SC-017-D5SC07248D-s001 [file SC-017-D5SC07248D-s001.pdf]

Supplementary Information (SI) for Chemical Science.
This journal is © The Royal Society of Chemistry 2025

Supplementary Information: Node-Equivariant Message Passing for Efficient and Accurate Machine Learning Interatomic Potentials

Yaolong Zhang* and Hua Guo

Department of Chemistry and Chemical Biology, Center for Computational Chemistry, University of New Mexico, Albuquerque, New Mexico 87131, USA

* Corresponding author, email: ylzhangch@unm.edu

Training details

Loss function. Networks are trained using a loss function based on a weighted sum of energy and forces:

$$\mathcal{L} = \lambda_E \sum_{i=1}^{N_b} \left(\frac{E_i^{\text{NN}} - E_i^{\text{DFT}}}{N_{atom}} \right)^2 + \lambda_F \sum_{i=1}^{N_b} \left\| \mathbf{F}_i^{\text{NN}} - \mathbf{F}_i^{\text{DFT}} \right\|^2 . \text{* MERGEFORMAT}$$

(15)

Here λ_E and λ_F denote the energy and force weights, respectively, which are hyperparameters. N_b is the batch size. We found that using a decaying force weight with learning rate decay—from approximately 10 to 0.1/1.0—while keeping the energy weight fixed at unity yields optimal test error. The only exception is 3BPA, for which we use a constant force weight of 0.2. E^{NN} (F^{NN}) and E^{DFT} (F^{DFT}) denote the energies (forces) predicted by the neural network (NN) and calculated using density functional theory (DFT), respectively. Our package also supports training of the stress tensor; however, this feature was not employed in the present work and is therefore not discussed here.

Network Architecture and Hyperparameters

3BPA. We employed a node-equivariant message passing (NEMP) model consisting of 3 message passing (MP) layers, each having 64 channels. To improve efficiency in each MP layer, we used different maximal angular momenta: L_{max}=3 (as in the Eq. (5) in main text) for evaluating spherical harmonics, and l_{max}=2 (the maximal value of l_f in Eq. (11) in the main text) for the tensor product in MP layer. The cutoff radius was set to 6.0 Å. The learning rate was initially warmed up from 0.01 to 0.2, then decayed by a factor of 0.5 whenever the validation loss did not decrease for 60 consecutive steps,

until reaching a minimum of 1×10^{-5}. The batch size was set to 1. The NN structure of F_{emb} comprised two ResNet blocks[1, 2], each with two layers of size 128×128 (denoted as 2×(128×128), used hereafter). Both F_{coeff} and $F_{readout}$ were implemented as linear connections to enhance transferability. F_{emb}, F_{coeff} and $F_{readout}$ denote the corresponding functions (neural network or linear layer) as described in the main text.

Liquid water. We employed an NEMP model with 3 MP layers, each having 8 channels. Both $L_{\max}$ and $l_{\max}$ were set to 2. The cutoff radius was 4.5 Å. The learning rate was initially warmed up from 0.001 to 0.01, then decayed by a factor of 0.035 whenever the validation loss did not decrease for 100 consecutive steps, until reaching a minimum of 1×10^{-5}. The batch size was set to 1. The NN structure of the F_{emb} was 2×(64×64), that of the F_{coeff} was 1×(32×32), and that of the $F_{readout}$ was 2×(32×32). For the NEMP-small model, the MP layers were reduced to 2, each having 4 channels. The F_{coeff} NN structure was reduced to 1×(12×12); all other settings remained the same as in the standard model.

ANI-1x. We employed an NEMP model with 3 MP layers, each having 32 channels. $L_{\max}$ was set to 3 and $l_{\max}$ were set to 2. The cutoff radius was 6.0 Å. The learning rate was initially warmed up from 0.001 to 0.01, then decayed by a factor of 0.1 whenever the validation loss did not decrease for 32 consecutive steps, until reaching a minimum of 1×10^{-5}. The batch size was set to 64. The NN structure of the F_{emb} was 2×(128×128), that of the F_{coeff} was 2×(128×128), and that of the $F_{readout}$ was 2×(256×256).

HME21. We employed an NEMP model with 3 MP layers, each having 16 channels. Both $L_{\max}$ and $l_{\max}$ were set to 2. The cutoff radius was 6.0 Å. The learning rate was

initially warmed up from 0.001 to 0.01, then decayed by a factor of 0.035 whenever the validation loss did not decrease for 50 consecutive steps, until reaching a minimum of 1×10^{-5}. The batch size was set to 2. The NN structure of the F_{emb} was 2×(128×128), that of the F_{coeff} was 2×(64×64), and that of the $F_{readout}$ was 2×(128×128).

EMLP. We employed an NEMP model with 3 MP layers, each having 16 channels. Both $L_{\max}$ and $l_{\max}$ were set to 2. The cutoff radius was 5.0 Å. The learning rate was initially warmed up from 0.001 to 0.01, then decayed by a factor of 0.5 whenever the validation loss did not decrease for 20 consecutive steps, until reaching a minimum of 1×10^{-5}. The batch size was set to 32. The NN structure of the F_{emb} was 2×(128×128), that of the F_{coeff} was 2×(64×64), and that of the $F_{readout}$ was 2×(128×128).

Reference:

1. K. He, X. Zhang, S. Ren and J. Sun, 2016 IEEE Conference on Computer Vision and Pattern Recognition (CVPR), 2016. **1512**, 770-778.
2. Y. Zhang, J. Xia and B. Jiang, *J. Chem. Phys.*, 2022, **156**, 114801.
